# Improved Shelf-Life and Consumer Acceptance of Fresh-Cut and Fried Potato Strips by an Edible Coating of Garden Cress Seed Mucilage

**DOI:** 10.3390/foods10071536

**Published:** 2021-07-02

**Authors:** Marwa R. Ali, Aditya Parmar, Gniewko Niedbała, Tomasz Wojciechowski, Ahmed Abou El-Yazied, Hany G. Abd El-Gawad, Nihal E. Nahhas, Mohamed F. M. Ibrahim, Mohamed M. El-Mogy

**Affiliations:** 1 Food Science Department, Faculty of Agriculture, Cairo University, Giza 12613, Egypt; 2 Natural Resources Institute, University of Greenwich, Central Avenue, Chatham Maritime, Kent ME4 4TB, UK; A.Parmar@gre.ac.uk; 3 Department of Biosystems Engineering, Faculty of Environmental and Mechanical Engineering, Poznań University of Life Sciences, Wojska Polskiego 50, 60-627 Poznań, Poland; gniewko.niedbala@up.poznan.pl (G.N.); tomasz.wojciechowski@up.poznan.pl (T.W.); 4 Department of Horticulture, Faculty of Agriculture, Ain Shams University, Cairo 11566, Egypt; ahmed_abdelhafez2@agr.asu.edu.eg (A.A.E.-Y.); hany_gamal2005@agr.asu.edu.eg (H.G.A.E.-G.); 5 Department of Botany and Microbiology, Faculty of Science, Alexandria University, Alexandria 21515, Egypt; nihal.elnahhas@alexu.edu.eg; 6 Department of Agricultural Botany, Faculty of Agriculture, Ain Shams University, Cairo 11566, Egypt; ibrahim_mfm@agr.asu.edu.eg; 7 Vegetable Crops Department, Faculty of Agriculture, Cairo University, Giza 12613, Egypt; elmogy@agr.cu.edu.eg

**Keywords:** *Lepidium sativum*, potato, browning index, oil uptake, antioxidant activity

## Abstract

Coatings that reduce the fat content of fried food are an alternate option to reach both health concerns and consumer demand. Mucilage of garden cress (*Lepidium sativum*) seed extract (MSE) was modified into an edible coating with or without ascorbic acid (AA) to coat fresh-cut potato strips during cold storage (5 °C and 95% RH for 12 days) and subsequent frying. Physical attributes such as color, weight loss, and texture of potato strips coated with MSE solutions with or without AA showed that coatings efficiently delayed browning, reduced weight loss, and maintained the texture during cold storage. Moreover, MSE with AA provided the most favorable results in terms of reduction in oil uptake. In addition, the total microbial count was lower for MSE-coated samples when compared to the control during the cold storage. MSE coating also performed well on sensory attributes, showing no off flavors or color changes. As a result, the edible coating of garden cress mucilage could be a promising application for extending shelf-life and reducing the oil uptake of fresh-cut potato strips.

## 1. Introduction

Potato *(Solanum tuberosum* L.) is one of the most popular crops globally. It is the fourth largest crop in production, after rice, wheat, and maize [1]. In 2019, and according to FAO statistics, the world production of potatoes was around 400 million tons from 19.25 million ha. Apart from its importance as a significant carbohydrate supplier, potato is an excellent source of several essential minerals, vitamins, dietary fiber, and antioxidants [2]. The demand for fresh-cut, minimal processed, and ready-to-eat fruits and vegetables, including potatoes, is increasing, particularly in urban areas due to the convenient nature of these products. During processing, the unit operations (peeling, cutting, and slicing) cause severe damage to living tissues, which increases the respiration rate, enzymatic browning, microbial spoilage, and water loss, resulting in quality losses and reduced shelf-life [3,4].

Enzymatic browning is one of the most prevalent causes of deterioration and loss of quality in processed potato products. Enzymatic browning negatively affects the sensory quality and nutritional components of potatoes [5]; it is triggered when phenolic compounds are leaked from damaged tissues and oxidized by polyphenol oxidase on the surface of fresh-cut potatoes [6]. Therefore, inhibiting enzymatic browning by various pre-treatments has been an important topic of research. Various pre-treatments have been studied for preventing enzymatic browning in potatoes, for example, dipping in hot water (55 °C) for 10 min [7], ultrasonic treatment [8], exogenous γ-aminobutyric acid (20 g L^−1^) application [9], and the application of 3-mercapto-2-butanol under the concentration of 25 μL L^−1^ at 5 °C [10]. Moreover, the application of antioxidants (oxalic and ascorbic acid) is well known to control enzymatic browning [11,12]. However, some of these anti-browning agents and treatments are generally not practical in industrial application due to their low customer acceptance, high cost, and negative impact on physicochemical properties.

Several dishes are prepared from potato in almost different cultural and geographical regions. However, one of the most common preparations is deep-fried potato chips and fries [13]. Consumer preferences are changing towards convenient food, as they look for healthier choices with low sugar and fat content [14]. Reducing oil uptake for fried foods by pre-drying before frying, frying under high temperature for a short time, and using edible coatings has been a subject of interest in the previous research [15]. The edible coating to reduce oil update in fried foods can provide a practical alternative for commercial applications. However, the research and development in the edible coating to reducing oil uptake are still in their infancy. This article contributes to developing edible coatings as a mainstream application to reduce the oil content of fried foods such as potato chips and fries.

*Lepidium sativum* is known as garden cress or pepper cress and belongs to the family *Bassicaceae* [16]. The various parts of the garden cress plant, such as seeds, leaves, or aerials, contain different phytochemicals (flavonoids, glycosides, alkaloid, and polyketides) and vitamins, minerals, proteins, fats, and carbohydrates. The principal constituents of garden cress are linolenic acid (33%) and oleic acid as the most essential fatty acids (23%), the principal sterol is β sitosterol (50%), and tocopherol (1.5–1.9 g kg^−1^) with c-tocopherol [17]. Due to these phytochemicals, garden cress is known for its various therapeutic and herbal effects [18]. Garden cress gum, categorized as a kind of water-soluble hydrocolloid, might be a potential raw material for preparing edible films and coatings. This is due to its physical, mechanical, optical, and barrier characteristics, which are essential in food packaging applications [19]. The biodegradable, edible nature of the garden cress seed gum coating and the seeds’ characterization (physical, microstructural, mechanical thermal characteristics) have been studied before [20]. A significant quality (microbiological, chemical, and sensorial) and shelf-life (cold storage) improvement was observed in shrimp samples coated with garden cress seed gum that contains 10% carvacrol [21]. The major advantages of edible films and coatings are biocompatibility, eco-friendliness (reducing 66% of total packaging wastes), low cost, and excellent barrier properties for gases, lipid, and aroma. Moreover, edible coatings can also be used as a carrier for foods additives and natural bioactive compounds such as vitamins, antioxidants, and antimicrobial compounds [3,22].

This study aimed to evaluate the effect of edible coating based on garden cress (*Lepidium sativum*) seed mucilage (gum) with or without ascorbic acid on the shelf life, microbial load, physical properties, oil uptake, and consumer acceptance of fresh-cut potato strips during cold storage at 5 °C for 12 days, which were subsequently fried into potato fries.

## 2. Materials and Methods 

### 2.1. Materials 

*Lepidium sativum* (Golden grass) seeds were purchased from the local market in Giza, Egypt. Methyl alcohol, gallic acid, sodium carbonate, 2,2-Diphenyl-1-picrylhydrazyl “DPPH” and Folin and Ciocalteu’s phenol reagent were purchased from Sigma-Aldrich (USA). Potato dextrose agar, plate count agar, and ethylene methylene blue agar were purchased from Oxoid Ltd. (Basingstoke, UK).

### 2.2. Water Extraction of Lepidium Sativum Seeds Mucilage

The mucilage was extracted according to Karazhiyan et al. [23] with some modifications. First, 50 g of seeds were soaked in 500 mL of distilled water for 12 h and followed by blending (Blender, Moulinex 400 W, Model: LM2420, French) for 15 min at 4000 RPM. Next, blended seeds were filtered under vacuum using the Buchner flask. The pH (pH meter: Jenway, model 3305, Dunmow, Essex, UK) of mucilage seed extract (MSE) was 4.7. For preparing the coating solution, 0.5% glycerol was added to seed mucilage extract and then pasteurized at 90 °C for 1 min.

### 2.3. Total Phenolic Compounds of Seedcake and MSE

The phenolic content in the fruit juices was estimated by the Folin–Ciocalteu method as described by Awad et al. [4] with some modifications. Five grams of homogenized *L. sativum* seedcake or 5 mL mucilage seed extract was extracted with 50 mL of methanol 80% in a conical flask with a shaker at 1000 rpm for 1 h at room temperature. The extract was then filtered with filter paper No 1; 0.5 mL of the extract was mixed with 2.5 mL of Folin–Ciocalteu reagent (1:10 with water) and, after 3 min, 2 mL of sodium carbonate (7.5%) was added. The absorbance was measured at 765 nm after 1 h of incubation in the dark at room temperature by a spectrophotometer (Unico UV-2000, UNICO company, Fairfield, NJ, USA). TPC was expressed as the gallic acid (GAE g kg^−1^) dry weight of seeds or 1 L of MSE.

### 2.4. Antioxidant Activity % of MSE 

The antioxidant activity of MSE was determined according to Ali and El Said [24]. First, 1 mL of the MSE was added to 3 mL of methanol and 1 mL of 2,2-diphenyl-1-picrylhydrazyl (DPPH) (0.024 g DPPH in 100 mL^−1^ of methanol). The mixture was incubated in the dark at room temperature for 30 min, followed by absorbance measurements at 517 nm. The antioxidant activity was expressed as % of activity (Equation (1)).

(1) $$Activity\;\left(\%\right)=\left[\frac{A\;control-A\;sample}{A\;control}\right]\times 100$$

*A control*: the absorbance of the control.

*A sample*: the absorbance of the sample.

### 2.5. Preparation of Coated Fresh-Cut Potato Strips

The potatoes were hand-peeled, cut into strips (1.0 × 1.0 × 8 cm) with a manual French fry cutter and washed with cold water to remove excess starch. The potato strips were then drained to reduce the water content on the surface. The samples were divided into 5 treatments as follow:

1. Control: the strips were dipped in distilled water for 5 min.
2. Ascorbic acid (AA): the strips were dipped in AA solution with a concentration of 500 ppm for 5 min.
3. Mucilage seed extract (MSE): the strips were dipped in MSE-coating solution for 5 min.
4. Mucilage seed extract with ascorbic acid (MSE + AA): the strips were dipped in MSE + AA (250 ppm) coating solution for 5 min.
5. Blanching: the strips were blanched for 2 min at 97 °C.

The strips were dipped at room temperature using gentle magnetic agitation (the ratio of grams of potato tissue to milliliters of the solution was 1:4). The strips were allowed to dry at ambient conditions and then placed in polypropylene trays covered with polypropylene pouches (each pouch contained 200 g potato strips (“approx. 20 potato strips”), which were closed thermally. The samples were stored at 5 °C and 95% RH for 12 days. The strips potato samples were taken at 4 d intervals for physical, chemical, and microbiological analysis. Specifically, we used 3 pouches as replicates for each treatment, so totally we prepared 9 pouches for each treatment.

### 2.6. Frying of Potato Strips

All samples were deep fried according to Reis et al. [25] in a deep frier (Orbit Pan 2000 Watts, Model: 2724297419595, Turkey) with a capacity of three liters of refined canola oil (1:6 strips: oil ratio) at 170 °C for 4 min. The oil was changed every time each batch was fried. Finally, the fried strips were allowed to cool and analyzed for oil uptake at room temperature.

### 2.7. Determination of Weight Loss, Texture, and Browning Index

Potato strips were weighed immediately after drying and at 0, 4, 8, and 12 days. The results are shown as the percentage weight loss compared to the initial fresh weight [26]. Ten potato strips from each treatment were used to determine texture using a digital penetrometer (PCE-PTR 200, PCE Americas Inc., Jupiter, FL, USA), and values are presented as Newtons (N). The browning index (BI) was calculated according to El-Mogy et al. [3] using Equation (2).

(2) $$BI=\frac{\left[100\;\left(x-0.31\right)\right]}{0.17}$$

where:

$$x=\frac{\left(a^{*}+1.75L^{*}\right)}{\left(5.645L^{*}+a^{*}-0.3012b^{*}\right)}$$

A Minolta colorimeter (Model CR-400 Chroma Meter, Konica Minolta, INC, Tokyo, Japan) was used to measure a* (color change from red to green), b* (color change from yellow to blue), and L* (lightness) values. A standard white calibration plate was used to calibrate the colorimeter [27].

### 2.8. Total Phenolic Compounds (TPC) of Potato Strips

The total phenolic compounds were determined according to the Folin–Ciocalteu spectrophotometric method described by Awad et al. [3] for potato strips, *L. sativum* seedcake and mucilage seed extract (MSE). Five grams of homogenized potato strips (interval during storage time) were extracted with 50 mL of 80% methanol in a conical flask with a shaker at 1000 rpm for 1 h at room temperature. The extract was then filtered with filter paper No 1., and 0.5 mL of potato extract was mixed with 2.5 mL of Folin–Ciocalteu reagent (1:10 with water), and after 3 min, 2 mL of sodium carbonate (7.5%) was added. The absorbance was measured at 765 nm after 1 h of incubation in the dark at room temperature. TPC was expressed as the gallic acid (GAE mg 100 g^−1^) fresh weight of potato strips.

### 2.9. Oil Uptake (OU)%

Fat content (FC)% of potato French fries were determined according to a Bligh and Dyer [28] extraction. The OU% in the coated potato French fries relative to the uncoated ones was expressed as in Equation (3):

(3) $$OU\;\left(\%\right)=\frac{FC_{coated}}{FC_{uncoated}}\times 100$$

In addition, the reduction of oil uptake was calculated by Equation (4).

(4) $$Oil\;uptake\;reduction\;\left(\%\right)=\frac{FC_{coated}-FC_{uncoated}}{FC_{coated}}\times 100$$

where *FC* = fat content (%).

### 2.10. Microbiological Analysis

Ten grams of potato strip samples were crushed and diluted (1:10 *w*/*v*) in 0.1% buffered peptone water, homogenized by hand massaging for 3 min, and serially diluted with buffered peptone water. The total count was determined using the standard plate count method described by Shehata et al. [29] using plate count agar media and incubating plates at 30 ± 1 °C for 48 h. Mold and yeast counts were performed in potato dextrose agar by incubation at 25–28 °C for 5–7 days. *Escherichia coli* was determined according to ISO 16654 [30]. Microbial counts were expressed as log CFU g^−1^ of tissue.

### 2.11. Statistical Analysis

The experiments were performed using a completely randomized design. R version 4.0.2 Statistical Package (Vienna, Austria) was used for data analysis. A two-way ANOVA (analysis of variance) for measures was conducted with holm-corrected LSD tests for CLD letters. In addition, a non-metric multidimensional scaling (NMDS) for sensory parameters was performed having the first two dimensions of meta-MDS (comm = ff, k = 3) (multidimensional global scaling using mono-MDS).

### 2.12. Sensory Analysis

Sensory characteristics including color, taste, odor, texture, and overall acceptability were evaluated for fresh cut and fried potato strips once immediately after treatment by 50 untrained panelists of the Food Science Department (35 females and 15 males, aged 22 to 45 y). A 9-point Hedonic scale (0–2 = dislike extremely, 3–4 = dislike slightly, 5 = fair, 6–8 = like moderately, and 9 = excellent) was utilized for this purpose [31]. The potato strip samples were served at room temperature on two plates: fresh cut and fried potato strips.

## 3. Results and Discussion 

### 3.1. Weight Loss, Texture and Browning Index

**Figure 1:**
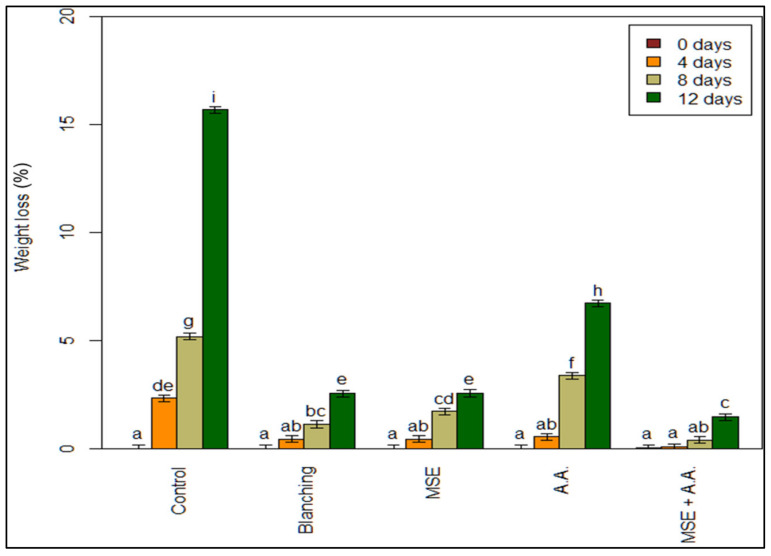
Effect of ascorbic acid (AA), mucilage seed extract (MSE), MSE + A.A, and blanching on weight loss (%) of fresh-cut potato strips. (Means with the same CLD letter are not statistically significantly different. Letters come from holm-corrected multiple comparisons across all times and treatments).

All treatments lost significantly less weight than the controls, starting from 4 days of storage until the end of the storage period (Figure 1). The lowest weight loss values were obtained from MSE + AA treatment. Similar effects of MSE + AA were shown previously on reducing weight loss in vegetables and fruits such as artichoke heads [3] and plums [32]. The beneficial effects of coating for decreasing weight loss might be due to adjusting the internal atmosphere and reduced respiration rates [3,29]. In addition, ascorbic acid contribution to the reduction in weight loss is possible due to the detoxification of active oxygen species [33].

**Figure 2:**
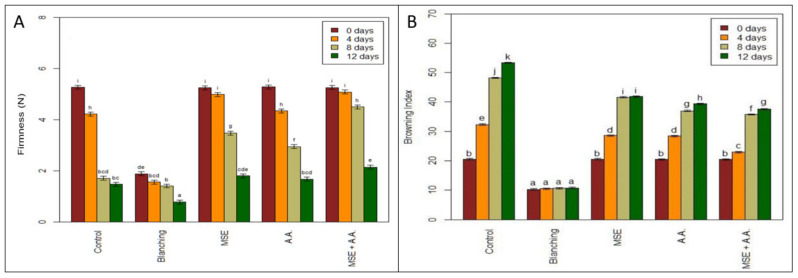
Effect of ascorbic acid (AA), mucilage seed extract (MSE), MSE + A.A, and blanching on firmness (N) (**A**) and browning index (**B**) of fresh-cut potato strips. (Means with the same CLD letter are not statistically significantly different. Letters come from a holm-corrected multiple comparisons, across all times and treatments).

As shown in Figure 2A, the texture of the control decreased with increasing storage time. The samples lost about 75% of their firmness after 12 days from the start of the storage.

Blanching led to lower firmness values than all treatments and the control starting from zero time until the end of storage. Moreover, the firmness of potato strips treated with MSE coating alone or with AA also decreased, but the decrease was significantly smaller than in other treatments. The firmness of fresh-cut fruits and vegetables is one of the most critical factors affecting the quality and shelf life [34]. Previous work reported that AA carried by chitosan as an edible coating layer significantly reduced firmness during refrigerated storage of plums fruits [32]. It was suggested that coating and AA application could reduce the main cell wall-degrading enzyme (such as polygalacturonase and pectin methylesterase) activity.

Enzymatic browning is one of the most prominent industrial problems in fresh-cut products due to the oxidation of phenolic compounds [3]. Our results indicated that browning increased by increasing the storage period (Figure 2B). Blanching showed the lowest browning index for overall storage periods compared to other treatments and control. A significantly lower browning index for the MSE + AA treatment was found during the whole storage period compared to the control. Although ascorbic acid as an anti-browning agent in fresh-cut vegetables has been reported previously [3], the effect is primarily due to the role of ascorbic acid as an oxygen scavenger that prevents oxidation by polyphenol oxidase [35].

### 3.2. Total Phenolic Compound and Antioxidant Activity of Seedcake and MSE

We determined the TPC in the dried garden cress seeds and in the seed mucilage after soaking in water to define the effect of the mucilage extraction method on the TPC content and to be sure of the quality of the garden cress that we used in this study. The aqueous extract contained 0.044 g GAE L^−1^, and seeds contained 0.441 g GAE kg^−1^ dry weight of TPC. Several studies have previously determined the TPC of *Lepidium sativum* seeds. Zia-Ul-Haq et al. [36] reported that the aqueous extracts of *Lepidium sativum* seeds contain 0.0120 g GAE kg^−1^. In addition, Chatoui et al. [17] reported that the TPC of *Lepidium sativum* seeds’ aqueous extract contains 0.62 g GAE kg^−1^ of seed extract.

Meanwhile, the obtained results showed a lower content of TPC in *L. sativum* seed extract. The TPC content varies depending on plant variety, agronomic practices, seed collection stage, and climatic and area geological condition of where seeds are harvested [37]. Rafińska et al. [38] indicated that extracting TPC of dried *L. sativum* seeds with water was an effective method due to the lowest level of interfering substances with high molecular masses. In addition, high hydrophilicity characterizes the phenolic compounds present in the seeds, possibly due to several hydroxyl groups.

The mucilage seed extract of *L. sativum* showed good antioxidant activity determined with DPPH, which recorded 90%. Chatoui et al. [17] noticed that the increase in TPC increases the antioxidant activity of the extract.

### 3.3. Changes in Total Phenolic Content of Fresh-Cut Potato Stripes

**Figure 3:**
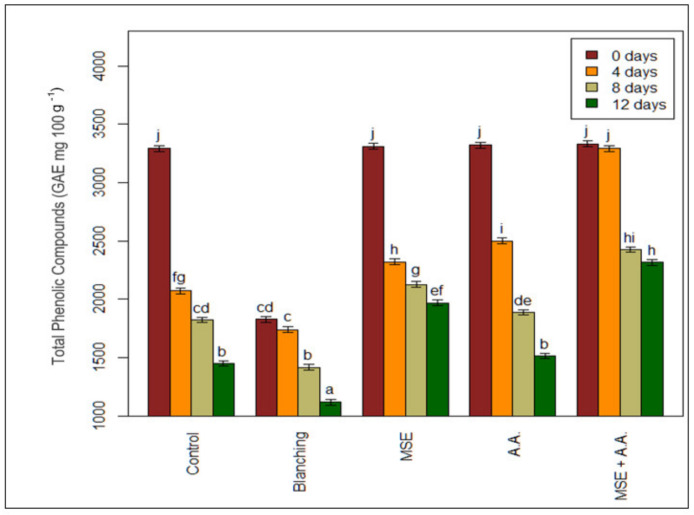
Effect of ascorbic acid (AA), mucilage seed extract (MSE), MSE + A.A, and blanching on total phenolic compounds (GAE mg 100 g^−1^) of fresh-cut potato strips. (Means with the same CLD letter are not statistically significantly different. Letters come from holm-corrected multiple comparisons, across all times and treatments).

For each treatment, the total phenolic content was determined in fresh-cut potato strips (Figure 3). The initial level of the phenolic compound ranged from 1827 to 3330 mg 100 g^−1^; there was no difference between all treatments, except for the blanched sample, which recorded the lowest initial levels of TPC (0.1827 mg 100 g^−1^ of potato strips). The phenolic compounds’ susceptibility to leaching from the plant tissue and degradation of heat-sensitive phenolic compounds can result in losses [39]. TPC contents of potato strips were significantly affected by the MSE treatment (Figure 3). MSE coating resulted in a 58% and 20% higher TPC content than the control sample (MSE + A.A.: 3291 mg 100 g^−1^, MSE: 2499 mg 100 g^−1^ fresh weight of potato strips). The most significant effect was observed when MSE was supplemented with A.A., where the final retention of the TPC after 12 days of storage was 2315 mg 100 g^−1^ fresh weight of potato strips. These results are following El-Mogy et al. [3], who indicated that edible coating with *Cordia* gum (edible polysaccharide coating with a high content of phenolic compounds), when supplemented with AA, had a significant positive effect on TPC of artichoke bottoms during cold storage at 4 °C for 9 days. However, the TPC decreased during the storage period in fresh-cut potato strips, possibly due to oxidative processes [40]. The higher TPC of coated samples suggests lower phenol oxidation than the control treatment, which can be due to edible coating preventing contact between food and oxygen, as well as the degradation of phenolic compounds [41].

### 3.4. Oil Uptake (OU)%

**Figure 4:**
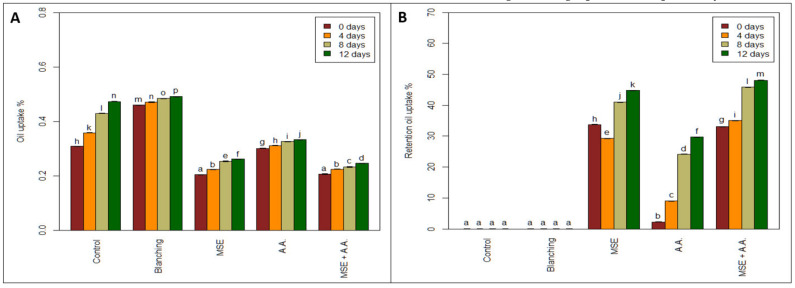
Effect of ascorbic acid (AA), mucilage seed extract (MSE), MSE + A.A, and blanching on oil uptake (**A**) and retention oil uptake (%) (**B**) of fresh-cut potato strips. (Means with the same CLD letter are not statistically significantly different. Letters come from holm-corrected multiple comparisons across all times and treatments.

Several studies have investigated the use of coating and blanching as a pre-treatment of frying for reducing the oil uptake [42,43]. All coating treatments (MSE and AA) significantly reduced OU% of fried potato strips compared to the uncoated sample (control, blanching) (Figure 4). As expected, the effect of coating showed that minimum oil content was related to MSE and MSE + AA (4.12, 4.08 g oil 100 g^−1^ potato), respectively.

In addition, the highest fat content was related to the blanched sample and control sample (non-coated) (9.19 and 6.16 g oil 100 g^−1^ potato, respectively). Previous studies have reported similar effects of blanching on oil uptakes [44,45]. Pedreschi and Moyano [45] noticed that blanching for high temperatures and short times (e.g., 97 °C, 2 min) before frying potato strips resulted in higher oil uptake than in control potato strips. This is because blanching involves the combined application of heat and water, which gelatinizes the starch on the surface of potato strips. This increase in oil content is undesirable for the acceptance of the product by the consumer.

The most important characteristics of edible coatings are oil barrier properties and flexibility because the volume of the food sample frequently changes during frying, and coating integrity may be compromised [42]. Consequently, hydrocolloidal coatings are used in various food applications, including adhesion, film shaping, thermal gelling, and non-charging properties. The form of the film properties of these hydrocolloids has stopped oil from being absorbed and has helped preserve the natural moisture content of the food. This may be the explanation for the deep frying of fried products using these hydrocolloids [46].

Therefore, the most effective coating treatments to reduce the percentage of oil uptake of fried potatoes were MSE + AA and MSE (33.08, 33.08%) at zero time. Moreover, there was no significant difference between MSE + AA and MSE at zero time or during the storage period of potato samples on oil content. The obtained results agreed with Garcia et al. [42], who found that the oil content of fried potatoes coated with 1% methylcellulose (MC) and 0.5% sorbitol was reduced in the range of 35–40%. This is related to the effective barrier properties of coating, which reduce oil uptake of fried potatoes. In addition, Mallikarjunan et al. [47] reported a protective layer formed on the surface of the fried products coated with cellulose derivatives during the initial stages of frying due to thermally induced gelation above 60°C. This layer could inhibit the transfer of fat and moisture between the sample and the frying oil.

To explain oil absorption, two main mechanisms are proposed as follows: condensation and capillary mechanisms; in both, inside the product, the oil penetrates through the pores. Therefore, the reduction in pore size and quantity can be due to the coating barrier limiting the oil uptake after frying. Thermo-gelling such as carboxymethyl cellulose (CMC), xanthan gum, and guar gum could lead to a more robust coating with low capillary pressures [13]. In addition, it is known that hydrocolloid treatment may alter the water-holding capacity and, consequently, prevent moisture replacement by oil [44].

### 3.5. Microbiological Analysis

**Figure 5:**
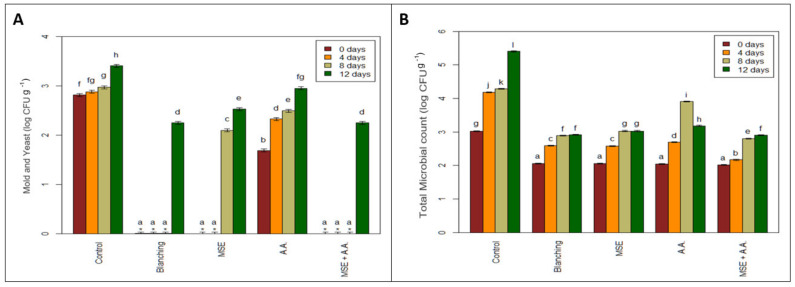
Effect of ascorbic acid (AA), mucilage seed extract (MSE), MSE + A.A, and blanching on mold and yeast (log CFU g^−1^) (**A**) and total microbial count (log CFU g^−1^) (**B**) of fresh-cut potato strips. (Means with the same CLD letter are not statistically significantly different. Letters come from holm-corrected multiple comparisons across all times and treatments.).

Figure 5 showed that the microbial load of the fresh-cut potato strips was affected by the cold storage duration at 5 °C for 12 days. All treatments were free from *E. coli* until the end of storage and had the same total microbial count, which ranged from 2.01 to 2.06 log CFU g^−1^ at zero time, except for the control, which had the highest count 3.02 log CFU g^−1^. At the end of storage, the lowest microbial count (2.91, 2.92 log CFU g^−1^) was found in both the MSE + AA and blanched samples, whereas the highest count (5.40 log CFU g^−1^) was observed in the control after 12 days of storage. In addition, at zero-time, the mold and yeast counts contained in all potato samples were below the detection limit (15 CFU g^−1^), except for the control and AA samples (2.81 and 1.69 log CFU g^−1^, respectively). The superior samples were MSE and MSE + AA, which remained free from mold and yeast growth after up to 8 d of cold storage.

There was no significant difference between all mold and yeast count treatments at the end of storage except for the control sample. On day 12, the control presented the highest count (3.40 log CFU g^−1^) while all other samples presented a lower count ranging from 2.25 to 2.95 log CFU g^−1^.

It is understood that microbiological safety is the most crucial factor in storing fresh-cut fruit and vegetables. Therefore, senescence and maturity reduce firmness, making the product more vulnerable to microbial attack [48]. In fresh-cut vegetables, both fungi and bacteria are significant causes of spoilage. The high-water activity of most vegetables and close neutral pH renders them adequate hosts for all kinds of microorganisms. However, the faster bacterial growth rate typically helps them to compete with the fungi more efficiently [49,50]. Cacace et al. [51] observed a more minor increase in the microbial count of fresh-cut potato treated with different chemicals such as erythorbic acid (5%) and citric acid (1%) with storage at 5 °C and reported a decrease in microbial growth with an increase in acidity. Dipping treatment using organic acids like ascorbic acid can also possess bactericidal properties [52]. The antimicrobial activity of organic acids is due to a decrease in environmental pH, disturbance of membrane transport and permeability, accumulation of anions, or a decrease in internal cellular pH due to the dissociation of acid from hydrogen ions.

Similarly, Licciardello et al. [53] showed that locust bean gum-based edible coating effectively reduced microbial growth. In addition, Barzegar et al. [54] noticed that *Lepidium sativum* seed mucilage-based edible coating containing 1.5% *Heracleum lasiopetalum* essential oil resulted in a significant increase in microbiological stability of the beef samples stored at 4 °C for 9 days, compared to the control. Such edible coating could prevent contact between food and oxygen. Therefore, incorporating mucilage of seeds such as *Lepidium sativum* can improve the barrier properties of the edible films against the transport of moisture and oxygen. Moreover, they can extend the shelf life of final products with their content of bioactive components (phenolic compounds), which have an antimicrobial effect [55]. However, the total microbial count and mold and yeast during cold storage is still well below the critical limits [56]. They mention that the critical limits for total microbial count and mold and yeast of vegetable were 108 CFU g^−1^ and 105 CFU g^−1^, respectively.

### 3.6. Sensory Evaluation

The most essential factors for consumer demand are sensory quality and nutrient contents of processed food products [57]. The ANOVA results showed no difference in the sensory appraisal of color, taste, odor, texture, and overall appearance of the samples among the different treatments after frying. However, before frying, the treatments showed an effect on the color and texture of the fresh potato strips, where the color of the control samples had the least rating, whereas all the treatments had no difference. For texture, blanched (before frying) samples had the least acceptability, and all other treatments, including the control, were the same. The color differences were related to the enzymatic and nonenzymatic browning (oxidation of phenolic compounds). It was expected that MSE might alter the taste of fried potato strips due to its pungent flavor [17]. However, the taste scores were highest for MSE samples. In the case of the blanched sample, a low score of taste and texture (of fresh and fried) regarding its high content of oil, as mentioned before, and heat treatment, which reduces firmness of the tissue. Reis et al. [24] reported that hydrocolloid coatings (CMC and xanthan gum) were used to improve the sensory attributes such as taste, texture, color, and appearance of French fries.

**Figure 6:**
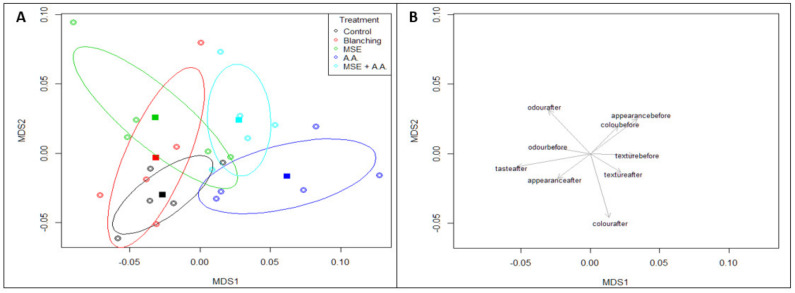
Non-metric multidimensional (NMDS) scaling for sensory parameters using mono-MDS. Circular points are replicates, solid square points are centroids for the data ellipses (by treatment) (**A**). Arrow plots showing weightings for sensory scores (**B**).

A non-metric multidimensional (NMDS) scaling for sensory parameters using mono-MDS is presented in Figure 6. Additionally, circular points are replicates, and solid squares are centroids for the data ellipses by treatment. In comparison, Figure 6B is an arrow plot, which shows that MSE and MSE + AA centroids lie at a higher level in the multidimensional spaces of the treatments, which means that the samples with MSE coatings showed higher rating results in the sensory evaluation. In Figure 6B, the weighing of each sensory attributes was calculated by the ordination techniques. The weightings for that attribute are further apart and the arrow from the center points higher, which shows that the total appearance before frying, color after frying, and odor after frying had the highest impact on the overall sensory evaluation results.

## 4. Conclusions

The novel method of utilizing garden cress seed extract into an edible coating to incorporate ascorbic acid help reduced the weight loss, browning index and preserve the firmness and total phenolic compound during storage of fresh-cut potatoes. Moreover, the coating helped reduce the oil uptake of the potato strips after frying. The sensory evaluation results showed that the sensory perception of the fried potato fries was enhanced using these edible coatings. Further research in commercial production and application of the garden cress seed extract is required for industrial application and testing its applicability to other fresh fruits and vegetable crops.

## Data Availability

Not applicable.
